# Radiotherapy travel times – is time running out in India?

**DOI:** 10.3332/ecancer.2022.ed122

**Published:** 2022-06-27

**Authors:** Aparna Gangopadhyay

**Affiliations:** Independent Practice, 377, M. B. Road, Panchanantala, Kolkata 700049, India

**Keywords:** radiotherapy, travel times, cancer control, treatment compliance

## Abstract

Inequitable radiotherapy availability in India leads to non-compliance in many cases, as patients need to travel long distances for treatment; this has long-term implications for achieving the United Nations sustainable development goals. Notably, the number of functional radiotherapy units in India is below the limit recommended by the World Health Organization, and most centers in this vast country are located in urban centers. This creates a serious barrier to accessibility for the socioeconomically disadvantaged sections of the rural population. Recent reports suggest that despite the availability of free treatments for a wide variety of cancers, many patients are non-compliant owing to the high costs incurred on travel to distant centers. In view of the current distribution of radiotherapy units, and the low ratio of radiotherapy units serving the vast population, distances traveled for radiotherapy are likely to have considerable impact on realization of the United Nations sustainable development goals. It is also likely to have considerable impact on the existing weak infrastructure of healthcare facilities, as poor cancer control will increase the need for palliative care and support, thereby further reducing resource allocation to cancer control. Policies directed towards reducing travel times for radiotherapy are currently lacking in India. However, this issue needs urgent consideration to ensure optimal utilization of available resources. Until measures to reduce travel time can be implemented, reducing travel-related patient distress may improve compliance in the short term; urgent measures in this regard will help achieve the targets of the United Nations sustainable development goals.

## Introduction

Distances traveled for cancer care have been found to be a potential barrier to cancer diagnosis and treatment, and have also been reported to be associated with patient outcomes [1-5]. Travelling longer distances for cancer care has also been linked to poorer survival among patients living in rural locations, despite early diagnosis [6].

India is a vast country, where approximately 65% of the population resides in rural areas. Most cancers continue to present in advanced stages, and require radiotherapy as part of treatment. However, radiotherapy availability is limited, and most functional units are located in urban areas, necessitating long-distance travel for treatment; this has considerable impact on treatment compliance.

This article describes the adverse impact of long-distance travel on treatment compliance, and therefore cancer control, in India; it also outlines possible interventions, that may help improve treatment compliance in both, the long and short term to help realization of the United Nations Sustainable Development Goals.

## Global goals for addressing cancer

Non-communicable diseases (NCDs) are a major contributor to global mortality, accounting for 7 of 10 deaths worldwide. As a considerable proportion of these deaths occur in individuals aged between 30 and 70 years of age, the United Nations Sustainable Development Goals aim to reduce premature mortality from 4 major NCDs by one third in this age group by the year 2030.

As one of the 4 major NCDs, cancer alone contributed to 10 million deaths in 2020 [7]. In order to address the overwhelming mortality burden from this disease, the World Cancer Declaration, which was launched under the leadership of the Union for International Cancer Control, was adopted to align it with the Global Action Plan for the Prevention and Control of Non-Communicable Diseases; the Declaration aims to achieve a 25% reduction in premature mortality from cancer by 2025, and has according set 9 targets to achieve this overarching goal. One of the targets is to improve accessibility to services across the cancer care continuum.

The World Health Assembly also passed a resolution in 2017 for cancer prevention and control in the context of an integrated approach (WHA70.12); this aimed to urge governments and the World Health Organization to accelerate action to achieve the targets specified.

### Global progress towards achieving the United Nations Sustainable Development Goals

Findings from a recent report suggest that only a few nations are on schedule to achieve the United Nations Sustainable Development Goals for preventing premature mortality from NCDs [8]. Global trends indicate that although premature mortality from NCDs is mostly on the decline worldwide, the pace of change in most nations is inappropriately slow for achieving the target. The report also suggests that the Coronavirus disease 2019 pandemic is seriously challenging the ability to meet the targets, and is increasing socioeconomic inequalities. Notably, most countries that were found to be on track to meet the goals were high-income nations.

In the context of cancer, the report suggests that although premature mortality from gastric cancer has declined rapidly, that from colorectal, liver, breast, and prostate cancer has declined more slowly; lung cancer among women was also found to be declining more slowly than necessary to meet the target. The most unfortunate finding was the increase in the risk of premature death from colorectal, liver, and prostate cancers in men, in more than half of the countries.

## The cancer problem in low-middle income countries

Low- and middle-income countries (LMICs) contribute to approximately 70% of deaths from cancer worldwide [9, 10]. Presentation at advanced stages and difficulties in access to healthcare facilities are major obstacles to early diagnosis and treatment in these settings. In addition, the availability of comprehensive treatment is as low as less than 15% in many of these countries, as opposed to more than 90% in high-income nations [11]. Owing to these difficulties, the prevention of premature cancer-related deaths is a considerable challenge in these settings. Indeed, recent data suggest that many LMICs have experienced stagnation or a minor increase in premature mortality from the disease [8].

Although cancer incidence is lower compared with high-income countries, deaths due to the disease are significantly higher in LMICs, particularly in those younger than 70 years. This premature loss of potentially productive individuals has considerable socioeconomic impact, and constitutes a major challenge to meeting the sustainable development goals.

### Radiotherapy in low-middle income countries

As part of multidisciplinary treatment, radiotherapy constitutes an indispensable component of cancer care. In view of its essential role in treating cancers across all stages and age groups, the importance of radiotherapy availability cannot be overemphasized. The weaknesses of healthcare systems in these settings, the rising burden of cancer, and financial constraints impact cancer control in these regions [12]. Data indicate that radiotherapy availability and accessibility are major issues in LMICs, and form a major barrier to effective treatment in many cases [13]. In particular, capacity building, maintenance of operability, and high population densities increase absolute radiotherapy need, which in turn translates to higher costs [14]. This poses a substantial challenge to improvement of radiotherapy availability in LMICs, leaving 90% of patients without access to radiotherapy [15]. In this context, a study found actual radiotherapy utilization rates in certain middle-income countries to be consistently lower than the optimal; this was particularly observed in countries with limited resources and large populations, with the median difference between optimal and actual utilization rates being as high as 47% [16].

## Radiotherapy accessibility in India

According to data from the Directory of Radiotherapy Centers, India has less than one radiotherapy machine per million individuals, with a majority of facilities offering treatment with megavoltage equipment [17]. Updated data from 2021 indicate that there are 427 centers with 669 megavoltage units and 317 brachytherapy units nationwide, in addition to a few advanced therapy units. The number of functional units is therefore below the limit recommended by the World Health Organization; in addition, most centers in this vast country are located in urban areas, which cannot be easily accessed by a large majority of the rural population. Socioeconomic disadvantages and financial toxicities, added to long waiting times resulting from overburdening of the available facilities, further compound difficulties in accessing treatment.

### Radiotherapy and the need for travel

Unlike other treatments for cancer, radiotherapy usually involves more frequent visits to the treatment facility. In order to improve the therapeutic ratio, radiotherapy treatments typically spread the total prescribed dose over a pre-determined number of fractions, which are administered as daily doses; most schedules deliver treatment five days a week. This necessitates regular visits, which often involve prolonged and exhausting journeys for those travelling for long distances. In patients with more advanced cancers, which are more common in LMICs, and those receiving concurrent chemotherapy, these journeys are particularly arduous.

### Travelling for radiotherapy in India

Recent reports suggest that despite the availability of free treatments for a wide variety of cancers, many patients are non-compliant owing to the high costs incurred on travel to distant centers [18]. Reports also indicate that the density and distribution of radiotherapy facilities in India lack uniformity, with certain regions having considerably lower numbers of treatment units compared to others in terms of the population served [19]. Interestingly, although most cancer facilities are located in urban areas, findings from a study performed at a rural cancer center indicate that over 60% of patients in that cohort who were non-compliant cited difficulties in travel; these patients were having to travel a distance of more than 100 km from home to hospital. Notably, these patients were receiving radiotherapy five days a week with weekly concurrent chemotherapy, and were further referred to a brachytherapy facility located over 500 km away on completion of teletherapy, for receiving three fractions of weekly high dose rate brachytherapy [20].

### Impact of long-distance travel for radiotherapy and implications in India

Numerous studies have demonstrated the impact of home-to-hospital distance on survival outcomes and quality of life among patients with cancer and survivors [21-23]. Although data on patient outcomes resulting from long-distance travel are largely unavailable in India, the impact of travel on treatment compliance is clear. In view of the current distribution of radiotherapy units, and the low ratio of radiotherapy units serving the vast population, distances travelled for radiotherapy are likely to have considerable impact on realization of the United Nations sustainable development goals. It is also likely to have considerable impact on the existing weak infrastructure of healthcare facilities, as poor cancer control will increase the need for palliative care and support, thereby further reducing resource allocation to cancer control.

## Urgent need for evaluation and monitoring of travel times

Although a few sporadic reports indicate the deleterious impact of prolonged travel times on treatment compliance, data on travel times to radiotherapy centers are lacking in India. In view of the far-reaching implications on cancer control in this vast country, travel times among patients attending various radiotherapy centers warrant urgent evaluation. Most studies consider a maximum travel time of within one hour as optimal [24, 25], while other reports suggest shorter times to be more appropriate [26]. In the absence of monitoring and relevant data availability, advisors for national radiotherapy policies are currently unable to regulate the installation of radiotherapy units based on regional needs; in addition to causing inequitable distribution of existing funding and resources, this is affecting optimal radiotherapy utilization nationwide.

## What can be done?

### What patients can do

In view of the considerable impact of non-compliance on radiotherapy outcomes and patient prognosis, it is essential that patients are educated on the detrimental effects of treatment delays and discontinuation. Patients may be encouraged to communicate travel related difficulties with their physicians to agree upon a more pragmatic fractionation schedule.

### How treatment centers can help

In view of the fractionated nature of radiation delivery, regular travel is an issue of particular concern to those travelling long distances; combined with financial toxicity and family burdens, this often leads to non-compliance in the Indian scenario.

Certain high volume centers prefer using hypofractionated schedules delivering equieffective doses for selected cases. This improves both, patient compliance and departmental throughput. However, owing to the lack of appropriate treatment planning infrastructure, advanced radiotherapy delivery equipment, appropriate funding, and associated resources, many centers are unable to deliver shorter and more convenient schedules in eligible cases; this leads to considerable loss of both, resources and time. Increasing volumes of data from international multicentric trials have established the safety and efficacy of certain hypofractionated schedules, and intraoperative and stereotactic radiotherapy delivery for various cancers commonly encountered in the radiotherapy departments across India [27-30]. Nevertheless, the lack of infrastructure, funding, and expertise are major hindrances to the implementation of these techniques. The availability of appropriate equipment, training, and incorporation of practices underlined in the Choosing Wisely initiative could make a major difference to patient convenience and departmental throughput.

### How policy-makers may contribute

Unlike many high income nations, India lacks a well-planned national intelligence network for monitoring and analyzing demographic data among patients undergoing radiotherapy. However, the launch of the Digital India programme has led to a paradigm shift in the availability of online infrastructure for all citizens. An increase in the use of digital-based platforms and a reduction in the urban-rural divide in terms of internet access are some of the major factors that policy-makers may use to their advantage. Policy-makers may consider initiating a national network to obtain patient travel data to improve planning for radiotherapy unit installation; this may reduce inequities in radiotherapy accessibility. Until a national network is initiated, it may be possible to estimate travel times from the home-to-hospital distance, as recorded during initial registration. In the initial stages, only information obtained from major cancer centers included in the National Cancer Grid may be collected and analyzed to evaluate trends; further implementation across other cancer centers may be performed stepwise to include all state and privately run satellite centers. Data from the extended network may then provide policy makers with a clearer understanding of regional needs, allowing more equitable distribution of funds and installation of centers.

### Improving compliance in the short term

In view of the long distances patients need to travel in order to access radiotherapy facilities, many residing at remote or rural areas arrange temporary accommodation near treatment facilities while undergoing radiotherapy; all expenses towards this arrangement are borne out-of-pocket, as governmental funding for free accommodation is currently unavailable. As nationwide data on travel for radiotherapy will not be immediately available for analysis, most patients will continue to arrange temporary accommodation near treatment facilities based on affordability. However, it is noteworthy that patients from below the national poverty line experience the highest levels of financial toxicity related to cancer care, and are often unable to pay for any additional expenses; this often leads to non-compliance. It would therefore be appropriate to ensure that all such patients are offered state-funded accommodation until home-to-radiotherapy facility distances in India are reduced, and travel times are reduced to acceptable limits nationwide.

### The road ahead

Artificial intelligence and data analysis are proving to be major game changers in various aspects of healthcare, in both high and low-middle income nations. As India continues to increase investment in this regard, further improvements are expected in the management of patient data, provision of care, and health equity. This will in turn increase the pace of change towards achieving the target of the sustainable development goals in this vast and diverse population. However, for this goal to be met, it is essential that the importance of travel times in successful radiotherapy treatment is recognized; it is the decision to walk that creates the path ahead.

## Funding

This research did not receive any specific grant from funding agencies in the public, commercial, or not-for-profit sectors.

## Conflicts of interest

None

## References

[1] Stoyanov DS, Conev NV, Donev IS (2022). Impact of travel burden on clinical outcomes in lung cancer. Support Care Cancer.

[2] Ambroggi M, Biasini C, Del Giovane C (2015). Distance as a barrier to cancer diagnosis and treatment: review of the literature. Oncologist.

[3] Loree JM, Javaheri KR, Lefresne SV (2017). Impact of travel distance and urban-rural status on the multidisciplinary management of rectal cancer. J Rural Health.

[4] Henley SJ, Anderson RN, Thomas CC (2017). Invasive cancer incidence, 2004–2013, and deaths, 2006–2015, in nonmetropolitan and metropolitan counties – United States. MMWR Surveill Summ.

[5] Zahnd W, Ganai S (2019). Access to cancer care in rural populations: barriers and solutions. ASCO Daily News.

[6] Turner M, Fielding S, Ong Y (2017). A cancer geography paradox? Poorer cancer outcomes with longer travelling times to healthcare facilities despite prompter diagnosis and treatment: a data-linkage study. Br J Cancer.

[7] Ferlay J, Ervik M, Lam F (2020). https://gco.iarc.fr/today.

[8] NCD Countdown 2030 collaborators (2020). NCD Countdown 2030: pathways to achieving Sustainable Development Goal target 3.4. Lancet.

[9] WHO International Agency for Research on Cancer (2020). Estimated number of deaths in 2020, all cancers, both sexes, all ages. Cancer Today.

[10] The Lancet GLOBOCAN 2018: counting the toll of cancer. Lancet.

[11] World Health Organization (2020). Assessing National Capacity for the Prevention and Control of Noncommunicable Diseases: Report of the 2019 Global Survey.

[12] Shah SC, Kayamba V, Peek RM (2019). Cancer control in low- and middle-income countries: is it time to consider screening?. J Glob Oncol.

[13] Grover S, Xu MJ, Yeager A (2015). A systematic review of radiotherapy capacity in low- and middle-income countries. Front Oncol.

[14] Zubizarreta E, Van Dyk J, Lievens Y (2017). Analysis of global radiotherapy needs and costs by geographic region and income level. Clin Oncol (R Coll Radiol).

[15] Abdel-Wahab M, Zubizarreta E, Polo A (2017). Improving quality and access to radiation therapy–an IAEA perspective. Semin Radiat Oncol.

[16] Rosenblatt E, Fidarova E, Zubizarreta EH (2018). Radiotherapy utilization in developing countries: an IAEA study. Radiother Oncol.

[17] IAEA (2021). Availability of Radiotherapy. Number of Radiotherapy Machine per Million Persons.

[18] Business Standard (2020). Few facilities, costly travel: Free cancer treatment, yet patients drop out. IndiaSpend.

[19] Munshi A, Ganesh T, Mohanti BK (2019). Radiotherapy in India: History, current scenario and proposed solutions. Indian J Cancer.

[20] Dutta S, Biswas N, Muhkherjee G (2013). Evaluation of socio-demographic factors for non-compliance to treatment in locally advanced cases of cancer cervix in a rural medical college hospital in India. Indian J Palliat Care.

[21] Thomas AA, Gallagher P, O’Céilleachair A (2015). Distance from treating hospital and colorectal cancer survivors’ quality of life: a gendered analysis. Support Care Cancer.

[22] Lee B, Goktepe O, Hay K (2014). Effect of place of residence and treatment on survival outcomes in patients with diffuse large B-cell lymphoma in British Columbia. Oncologist.

[23] Baade PD, Dasgupta P, Aitken JF (2011). Distance to the closest radiotherapy facility and survival after a diagnosis of rectal cancer in Queensland. Med J Aust.

[24] Onega T, Duell EJ, Shi X (2008). Geographic access to cancer care in the U.S. Cancer.

[25] Rocque GB, Williams CP, Miller HD (2019). Impact of travel time on health care costs and resource use by phase of care for older patients with cancer. J Clin Oncol.

[26] Oxford Cancer Intelligence Unit (2012). National Cancer Intelligence Network Travel times and distances to radiotherapy centres for head and neck cancer patients in England (2006–2008). http://www.ncin.org.uk/cancer_type_and_topic_specific_work/cancer_type_specific_work/head_and_neck_cancers/head_and_neck_cancer_hub/resources.

[27] Haviland JS, Owen JR, Dewar JA (2013). The UK Standardisation of Breast Radiotherapy (START) trials of radiotherapy hypofractionation for treatment of early breast cancer: 10-year follow-up results of two randomised controlled trials. Lancet Oncol.

[28] Vaidya J S, Bulsara M, Baum M (2020). Long term survival and local control outcomes from single dose targeted intraoperative radiotherapy during lumpectomy (TARGIT-IORT) for early breast cancer: TARGIT – a randomised clinical trial. BMJ.

[29] Bensaleh S, Bezak E, Borg M (2009). Review of MammoSite brachytherapy: advantages, disadvantages and clinical outcomes. Acta Oncol.

[30] Ball D, Mai GT, Vinod S (2019). Stereotactic ablative radiotherapy versus standard radiotherapy in stage 1 non-small-cell lung cancer (TROG 09.02 CHISEL): a phase 3, open-label, randomised controlled trial. Lancet Oncol.

